# Erratum and republication: Identification of a new Salmonella serovar – Salmonella Weitmar (8:z41:1,5)

**DOI:** 10.3205/id000103

**Published:** 2026-01-14

**Authors:** Carlos José Téllez-Castillo, Ann-Katrin Rekendt, Susanne Kollberg-Dix, Laura Pra-Mio, Claas Scharmann

**Affiliations:** 1 Praxis für Labormedizin und Mikrobiologie, Bochum, Germany

Erratum for: Téllez-Castillo CJ, Rekendt AK, Kollberg-Dix S, Pra-Mio L, Scharmann C. Identification of a new Salmonella serovar – Salmonella Scharmann (8:z41:1,5). GMS Infect Dis. 2025;13:Doc08. DOI: 10.3205/id000098

## Notification

Following the publication of the article

Téllez-Castillo CJ, Rekendt AK, Kollberg-Dix S, Pra-Mio L, Scharmann C. Identification of a new Salmonella serovar – Salmonella Scharmann (8:z41:1,5). GMS Infect Dis. 2025;13:Doc08. DOI: 10.3205/id000098

the serovar name *Salmonella Scharmann* was formally changed to *Salmonella Weitmar* by the WHO Collaborating Centre for Reference and Research on Salmonella (Institut Pasteur, Paris, France). A corrected version of the article has therefore been republished; the scientific content, data, and conclusions remain unchanged:

Téllez-Castillo CJ, Rekendt AK, Kollberg-Dix S, Pra-Mio L, Scharmann C. Identification of a new Salmonella serovar – Salmonella Weitmar (8:z41:1,5). GMS Infect Dis. 2026;14:Doc02. DOI: 10.3205/id000102

## Explanation

In the originally published version of article, the serovar name *Salmonella Scharmann* was used. At the time of submission and publication, this designation had been officially confirmed by the relevant reference authorities. Following publication, the WHO Collaborating Centre for Reference and Research on *Salmonella* (Institut Pasteur, Paris, France), via the Institute for Hygiene and Environment (Hamburg, Germany), clarified that the designation “Scharmann” is considered a personal name and therefore does not comply with current international nomenclature rules. The WHO Reference Centre has since officially confirmed the alternative geographical designation *Salmonella Weitmar*.

Accordingly, the serovar name in the article, in association with the antigenic formula (8:z41:1,5), has been formally changed from *Salmonella Scharmann* to *Salmonella Weitmar*. This correction does not affect the scientific content, data, or conclusions of the article.

## Notes

### Author’s ORCID

Carlos José Téllez-Castillo: 0000-0003-3722-2830

### Competing interests

The authors declare that they have no competing interests.

